# Validation of a Spanish-language scale for evaluating perceived quality of care of medical abortions before 9 weeks gestation

**DOI:** 10.1186/s12905-022-01763-5

**Published:** 2022-05-28

**Authors:** Rosa Cabedo-Ferreiro, Maria-Mercedes Vicente-Hernández, Josep-Maria Manresa-Domínguez, Miriam Gómez-Masvidal, Laura Montero-Pons, Azahara Reyes-Lacalle, Gemma Falguera-Puig

**Affiliations:** 1 Atenció a la Salut Sexual i Reproductiva, Institut Català de la Salut, Granollers, Barcelona, Spain; 2 Atenció a la Salut Sexual i Reproductiva, Institut Català de la Salut, Sant Adrià de Besòs, Barcelona, Spain; 3 Unitat de Suport a la Recerca Metropolitana Nord, Institut Universitari de Investigació en Atenció Primaria (IDIAPJGol), Cerdanyola, Barcelona, Spain; 4 Departament d’Infermeria, Universitat Autònoma de Barcelona, Cerdanyola, Barcelona, Spain; 5 Atenció a la Salut Sexual i Reproductiva, Institut Català de la Salut, Mataró, Barcelona, Spain; 6 Atenció a la Salut Sexual i Reproductiva, Institut Català de la Salut, Santa Coloma de Gramenet, Barcelona, Spain; 7 Atenció a la Salut Sexual i Reproductiva, Institut Català de la Salut, Sabadell, Barcelona, Spain; 8 GRASSIR Research Group, IDIAPJGol, Generalitat de Catalunya (SGR 2014-2016), Barcelona, Spain; 9 Sabadell, Spain

**Keywords:** Service quality, Medical abortion, Induced abortion, Satisfaction, Healthcare quality, Validation studies

## Abstract

**Background:**

Spanish Organic Law 2/2010 legalizes abortion within 14 weeks of gestation. Medical abortion with mifepristone and misoprostol is around 97% effective and is offered at primary care centers during the first 9 weeks of gestation. It consists of the administration of 200 mg of mifepristone by a healthcare professional and of the self-administration 800 mg of misoprostol by the patient at home, along with prescribed analgesics. However, the quality of this process as perceived by patients has never been assessed. This study aims to validate a scale designed to assess the perceived quality of the entire process, structure and results of at-home medical abortion.

**Methods:**

Validation study of a Spanish adaptation of the SERVPERF scale. In total, 289 patients completed a self-administered questionnaire consisting of 26 items previously evaluated by a group of experts. A re-test was performed on 53 of these patients 15 days later to assess interobserver consistency.

**Results:**

The highest non-response rate for any single item was 2.1%. The floor effect was 26% and the ceiling effect did not surpass 83%. The linearly weighted Kappa coefficient was good to excellent, in general. An exploratory factor analysis was performed with Varimax rotation, obtaining a total of 7 dimensions that explain 65.9% of the variability. The internal consistency (Cronbach's alpha) for all items was 0.862.

**Conclusion:**

This psychometric instrument is valid and reliable for assessing the quality of care of medical abortion. Medical abortion is efficient, effective and eliminates the need for hospital care, anesthesia and surgical risk. However, user satisfaction has yet to be determined. This study offers a validated scale to assess perceived quality of care, their quality experience and person-centered care for abortion as a fundamental part of overall service quality as a fundamental part of overall service quality.

## Introduction

In Spain, there were 95,917 abortions in 2018, which represents a rate of 11.12‰ of women of reproductive age. In our sphere of influence, the Barcelona North Metropolitan Area, 1,995 medical abortions (MA) were performed, of which 90% were complete expulsions and 3% required curettage [1, 2].

Abortion within 14 weeks gestation (WG) was legalized under Spanish Organic Law 2/2010 on Sexual and Reproductive Health and Abortions[3]. It is offered at the Sexual and Reproductive Health Care Centers (ASSIR) of the Primary Care (PC) service. This guarantees continuity of care and proximity to the population, as recommended by the World Health Organization (WHO) [4]. MA is carried out before 9 WG. It consists of the self-administration of 200 mg of mifepristone and 800 mg of misoprostol by the patient at home, along with prescribed analgesics. Patient progress is monitored and effective contraceptive counseling (aimed at allowing women to make a free and well-informed decision when choosing an effective contraceptive method and then using it properly) is provided as well [4].

MA haves significantly changed sexual and reproductive health care, resulting in the creation of specific visits aimed at interrupting women’s daily life as little as possible. The process has very high efficiency rates (99.7%) [5].

In recent decades, health organizations have worked towards continuous quality improvement, not just by evaluating the quality of processes, safety and results, but also taking into account patients, their service quality experience and perceived satisfaction [6].

In this regard, there are two quality improvement plans in place in Catalonia: a plan for the management of excellence and safety (Health Plan for Catalonia 2016–2020) [7] and a satisfaction survey plan (PLAENSA©) [6]. However, no data has been collected on the quality of the MA process.

Many studies have evaluated health services from the angle of perceived quality of care [8–10].To this end, quantitative quality measurement tools have been created, including the SERVQUAL [11, 12] and SERVPERF (SERVice-PERFormance) scales, which are based on customer perception. The SERVPERF survey [12] comprises 22 perceptions items divided into 5 quality dimensions. While this scale has been used to evaluate commercial services [13–16], it is also suitable for use in healthcare, having been employed to assess public health in Malaga [17], a health center in La Coruña [18],in an emergency service in Chile [17] and in women giving birth in Peru [19]. Such usage proved to be reliable and valid.

Patient satisfaction has been analyzed in studies comparing surgical and medical abortion methods and different family medicine centers where abortions were performed [20]. Mc Lemore and Wu studied the quality of abortion in the United States, identifying the issues that most concerned women via questionnaires of their own creation [21, 22].

The concept of perceived quality of care represents a significant methodological shift since the assessment of the quality of service is based on subjective criteria [18].There is consensus in the scientific community that at least the following aspects must be measured: (1) technical quality, (2) aspects related to the interpersonal relationship established during the process, and (3) the context in which the health service is provided [18]. This study was developed based on these three premises.

This study aims to validate a specific tool designed based on the SERVPERF model to measure quality of the care process, structure and results as perceived by patients who have a MA through PC.

## Materials and methods

### Study design

We conducted a validation study of a Spanish adaptation of the SERVPERF scale to assess quality as perceived by women who requested a MA.

The target population was women aged 16–49 years old who requested a MA at 7 ASSIR (public centers of the Catalan Institute of Health) in the Barcelona North Metropolitan Health Area, which has an assigned population of 341,511 women. Patients were excluded from the study if they were not proficient in Spanish, exceeded 9 WG; opted for surgical abortion, decided to continue the pregnancy or had a miscarriage.

To validate a questionnaire, 5–10 participants per item are needed [23]. Assuming that the participant dropout rate could reach 10%, a minimum of 290 women was required.

Over the course of 2019, the patients were recruited consecutively in proportion to the population attended to at each ASSIR center. If they met the criteria, they were informed of the study, and if they agreed to participate, they signed an informed consent form.

The project was authorized by the Research Ethics Committee of the Institute for Primary Health Care Research Jordi Gol (IDIAPJGol) under code P15/109.

### Description and administration of the questionnaire

A printed, self-administered questionnaire was designed, consisting of two parts:

1. During patients’ initial visit, sociodemographic information was collected: age, education attainment, living situation, social support, place of birth and year of arrival, employment situation, and obstetric history (number of abortions and living children). Data related to the MA process was also collected during this part of the questionnaire: gestational age, emergency contraception and use and type of contraception.
2. The second part of the questionnaire focused on the quality of the MA process . It consisted of 26 items, of which 20 were adapted from the SERVPERF scale and 6 corresponded to specific aspects of the MA process: perception of pain, intensity of bleeding, impact, and feeling judged (Table 1).
  Responses were ordinal, scored on a scale of 1–5 and included drawings of faces for easy interpretation. Items 1–18 focused on the care received. Items 19–24 addressed how the respondent was affected by the process, and items 25 and 26 asked whether they would recommend this method of abortion (Table 1) [23].
  The questionnaire was given to patients to complete on their own after the MA, at the last visit of the process.

**Table 1:** Items on the adapted SERVPERF questionnaire to determine the satisfaction of 289 patients who had a medical abortion

Item	Description	Very poor/poor/normal/good/excellent				
		1	2	3	4	5
P01	Qualification of the health professionals (abilities, experience, knowledge)					
P02	Sense of trust transmitted by the health professionals					
P03	Clarity of the information provided					
P04	Kindness shown by the health professionals					
P05	Interest shown by the health professionals in solving your problems during the process (questions answered, management, monitoring)					
P06	Amount of time dedicated to you by the health professionals					
P07	Health professionals’ appearance (personal hygiene)					
P08	Willingness of the administrative staff to provide immediate service					
P09	Training of the administrative staff					
p10	Coordination between the fields of healthcare and professional levels (primary care, sexual health, hospital)					
p11	Amount of information provided about the process (documents, procedures, possible side effects)					
p12	Aesthetics of the healthcare facilities					
p13	Ease of the procedures/paperwork					
p14	Ease of accessing the service (timetable, access to the center, parking, public transport)					
p15	Amount of time until first visit					
p16	Amount of time from first visit until the abortion					
p17	Information provided to prevent future pregnancies (contraceptive methods)					
p18	Information provided about subsequent psychological monitoring and resources					

		Excessive/moderate/tolerable/slight/imperceptible				
		1	2	3	4	5
p19	Pain experienced during the abortion					
p20	Level of anxiety experienced during the abortion					
p21	Amount of bleeding during the abortion					
p22	Impact of the abortion process on family relationships					
p23	Impact of the abortion on your life					
p24	Feeling judged by the staff					

		*Definitely not/…/absolutely*				
		1	2	3	4	5
p25	Would you return to the same health center if necessary?					
p26	If your friend were in the same situation, would you recommend a medical abortion?					

### Questionnaire validation

For the descriptive analysis, the qualitative variables were summarized with their absolute and relative frequencies, and the continuous variables with their mean and standard deviation.

### Viability

A pilot test was carried out to detect problems related to item comprehension, the Likert scale and any logistical issues with the study. It was performed on 24 patients, three by ASSIR centers. The mean response time for the questionnaire was 15 min.

After reviewing the results, the definitive questionnaire was designed on paper and later digitized using an optical reader (Teleform®).

### Metric characteristics

Lost records, floor and ceiling effects, and minimum and maximum response scores were summarized for each item.

### Content validity

The English version of the original SERVPERF questionnaire [12] was translated into Spanish and adapted to our area of interest with the help of two native Spanish-speaking translators who were proficient in English, thus obtaining a definitive version.

To formulate the questions of the adapted questionnaire, eight external experts were consulted, including gynecologists, midwives, and administrative staff. This group of experts suggested adding two items to assess the information provided to prevent future pregnancies and subsequent psychological monitoring and resources. The difficulty of assessing the presentation and physical appearance of the staff items was also discussed. They were combined into a single item called "appearance (personal hygiene)"so as to avoid assessing fashion choices, hairstyle, etc. The items "interest" and "willingness to solve…" were also combined into a single item as they were considered repetitive. The item "feeling judged by the staff" was added to address the ethical connotation of abortion. Lastly, the question "would you recommend this healthcare to a friend?" was changed to "would you recommend amedical abortion?" to switch the focus to the abortion method and avoid referring to abortion as a decision, so as not to broach ethical judgments.

### Construct validity

A factorial analysis with Varimax rotation was performed to determine the dimensions in which the items were grouped. The Kaiser criterion with a saturation value > 1 was used to identify the factors (dimensions) and explained variance. Saturation values > 0.40 were considered for each factor. The internal consistency of each resulting dimension was analyzed (Cronbach's alpha).

### Reliability

The internal consistency of the questionnaire was assessed with Cronbach’s alpha value and test–retest agreement with the linearly weighted Kappa coefficient.

To determine test–retest reliability, 50 women were contacted by phone to respond to the questionnaire a second time 15 to 21 days later.

## Results

### Description of the participants

Table 1 shows the items adapted from the questionnaire to validate.

**Fig. 1:**
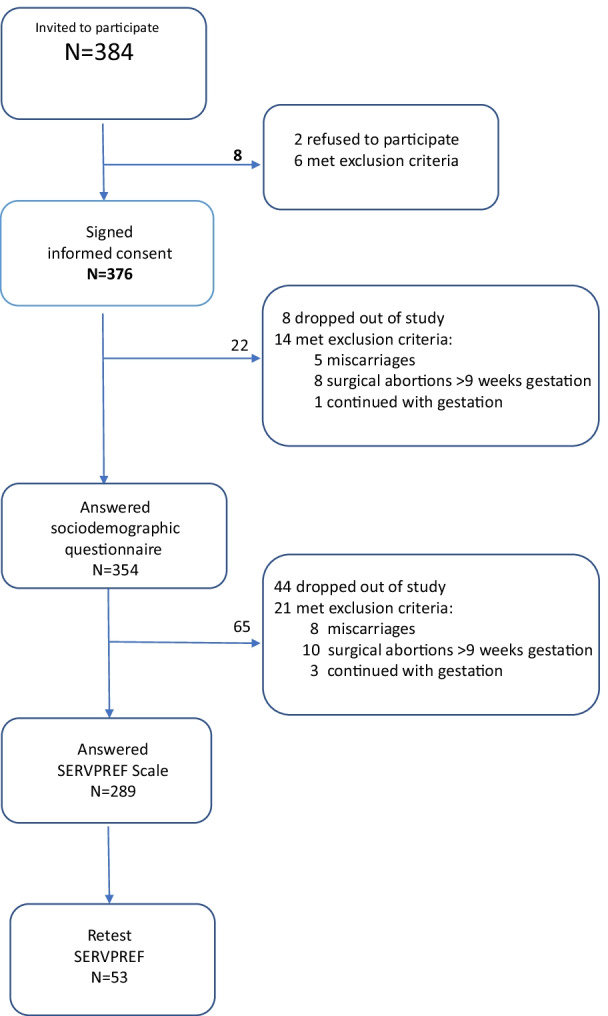
Participation flow chart. *Note*: This diagram shows the flow of participants and the reasons for the loss of participants

A total of 376 women were recruited, of which 354 continued with the MA process; 65 were removed for presenting exclusion criteria; 289 patients completed the study, while 44 (13.2%) dropped out (Fig. 1).

The mean age of participants was 29.5 (SD7.3, range 16–45) and mean gestational age was 6.3 weeks (SD 1.1); 78.7% had completed compulsory secondary education or high school/vocational school. Most participants were from Europe (70.5%), followed by Central and South America (23.2%); 74.4% had a paid job; 86.9% lived with their partner or family and 13.1% lived alone or in a shared flat. Social support was provided by participants’ partners (68.2%), followed by family (51.3%), and 4.9% reported not having social support (Table2).

**Table 2:** Sociodemographic characteristics of study participants and dropouts

Variable	Dropouts (n = 44)	Participants (n = 289)	*p*
Age	29.07 (10.0)	29.5 (7.3)	0.740
Gestational age	6.25 (1.1)	6.28 (1.1)	0.861
*Education attainment*			0.316
No studies or incomplete	2 (4.5%)	11 (3.8%)	
Compulsory education	8 (18.2%)	91 (31.7%)	
High school/vocational school	26 (59.1%)	135 (47.0%)	
University	8 (18.2%)	50 (17.4%)	
*Place of birth*			0.285
Europe (including Spain)	27 (61.4%)	201 (70.5%)	
Central and South America	15 (34.1%)	66 (23.2%)	
Morocco and the rest of Africa	2 (4.5%)	18 (6.3%)	
Paid employment	22 (66.7%)	189 (74.4%)	0.343
*Living situation*			0.662
Alone	6 (14.0%)	23 (8.1%)	
With partner	113 (30.2%)	93 (32.7%)	
With family	22 (51.2%)	154 (54.2%)	
In a shared flat	2 (4.7%)	14 (4.9%)	
*Social support*			
Partner	24 (57.1%)	187 (68.2%)	0.165
Family	24 (57.1%)	138 (51.3%)	0.481
Friends	15 (35.7%)	70 (26.1%)	0.195
No social support	4 (9.5%)	13 (4.9%)	0.219
*Number of previous abortions*			0.781
0	25 (56.8%)	154 (54.4%)	
1	12 (27.3%)	71 (25.1%)	
2	3 (6.8%)	34 (12.0%)	
3	2 (4.5%)	17 (6.0%)	
≥ 4	2 (4.5%)	7 (2.5%)	
*Number of living children*			0.998
None	22 (50.0%)	139 (49.1%)	
1	10 (22.7%)	67 (23.7%)	
2	9 (20.5%)	60 (21.2%)	
3	2 (4.5%)	10 (3.5%)	
≥ 4	1 (2.3%)	7 (2.5%)	
Use of contraception	27 (64.3%)	162 (58.9%)	0.508
*Type of contraception*			< 0.001
Condom	14 (51.9%)	110 (67.9%)	
Hormonal	8 (29.6%)	52 (32.1%)	
IUD	3 (11.1%)	0	
Emergency contraception	2 (7.4%)	26 (10.2%)	0.537

As for previous abortions, 45.6% of participants reported having had one or more; 49.1% did not have children; 58.9% reported using some method of contraception and 10.2% used the emergency contraceptive pill in that cycle. The condom was the most commonly used method (67.9%), followed by hormonal contraception (32.1%).

Patients who dropped out did not present significant differences, except for lower condom use (51.9% vs 67.9%) and greater use of hormonal methods or IUDs (40.7%).

### Questionnaire validation

The instrument presented the full range of potential responses to the items and non-response did not exceed 2.1% for any item. The floor effect was very low (0.3–26%); the ceiling effect was higher but did not exceed 83% (Table 3).

**Table 3:** Description of the responses for each questionnaire item

Item	(Min.–Max.)	Floor effect	Ceiling effect	Missing records	Mean (SD)
p1	(3–5)	13 (4.5%)	206 (71.3%)	4 (1.4%)	4.7 (0.56)
p2	(2–5)	3 (1.0%)	217 (75.1%)	0 (0.0%)	4.7 (0.61)
p3	(2–5)	2 (0.7%)	216 (74.7%)	2 (0.7%)	4.7 (0.55)
p4	(2–5)	3 (1.0%)	228 (78.9%)	2 (0.7%)	4.7 (0.59)
p5	(2–5)	2 (0.7%)	211 (73.0%)	0 (0.0%)	4.7 (0.57)
p6	(2–5)	2 (0.7%)	176 (60.9%)	1 (0.3%)	4.6 (0.62)
p7	(3–5)	7 (2.4%)	228 (78.9%)	6 (2.1%)	4.8 (0.47)
p8	(1–5)	1 (0.3%)	170 (58.8%)	1 (0.3%)	4.4 (0.79)
p9	(1–5)	2 (0.7%)	156 (54.0%)	2 (0.7%)	4.4 (0.79)
p10	(2–5)	4 (1.4%)	144 (49.8%)	2 (0.7%)	4.4 (0.75)
p11	(2–5)	3 (1.0%)	205 (70.9%)	0 (0.0%)	4.6 (0.62)
p12	(2–5)	6 (2.1%)	117 (40.5%)	2 (0.7%)	4.2 (0.80)
p13	(3–5)	27 (9.3%)	182 (63.0%)	1 (0.3%)	4.5 (0.66)
p14	(1–5)	2 (0.7%)	136 (47.1%)	1 (0.3%)	4.2 (0.86)
p15	(1–5)	1 (0.3%)	148 (51.2%)	1 (0.3%)	4.3 (0.81)
p16	(1–5)	3 (1.0%)	151 (52.2%)	2 (0.7%)	4.3 (0.87)
p17	(1–5)	1 (0.3%)	194 (67.1%)	1 (0.3%)	4.6 (0.67)
p18	(1–5)	2 (0.7%)	149 (51.6%)	1 (0.3%)	4.3 (0.85)
p19	(1–5)	63 (21.8%)	25 (8.7%)	3 (1.0%)	2.6 (1.21)
p20	(1–5)	34 (11.8%)	38 (13.1%)	4 (1.4%)	3.0 (1.22)
p21	(1–5)	75 (26.0%)	7 (2.4%)	3 (1.0%)	2.2 (0.98)
p22	(1–5)	15 (5.2%)	96 (33.2%)	5 (1.7%)	3.6 (1.23)
p23	(1–5)	20 (6.9%)	61 (21.1%)	6 (2.1%)	3.3 (1.22)
p24	(1–5)	8 (2.8%)	219 (75.8%)	6 (2.1%)	4.6 (0.92)
p25	(1–5)	2 (0.7%)	241 (83.4%)	2 (0.7%)	4.8 (0.58)
p26	(1–5)	5 (1.7%)	205 (70.9%)	3 (1.0%)	4.5 (0.90)

*Min.–Max*. Minimum and maximum values obtained for each item; *SD* Standard deviation

All items had a mean score of over 4 points, except items 19 to 23, which ranged from 2 to 4 points.

To determine test–retest reliability, 53 women answered the questionnaire a second time 15 to 21 days later (Fig. 1). The linearly weighted Kappa coefficient was good to excellent in general, moderate for items 6, 8 and 11, and low for item 17 (Table 4).

**Table 4:** Linearly weighted Kappa coefficient to evaluate intraobserver agreement (test–retest) for each item and overall

Item	Linearly weighted Kappa coefficient (CI 95%) (N = 53)	*p*
P01	0.715 (0.52–0.91)	0.000
P02	0.709 (0.50–0.92)	0.000
P03	0.810 (0.67–0.95)	0.000
P04	0.744 (0.56–0.93)	0.000
P05	0.691 (0.52–0.86)	0.000
P06	0.480 (0.28–0.68)	0.000
P07	0.627 (0.42–0.83)	0.000
P08	0.413 (0.19–0.64)	0.000
P09	0.756 (0.61–0.90)	0.000
P10	0.657 (0.50–0.81)	0.000
P11	0.460 (0.25–0.67)	0.000
P12	0.655 (0.51–0.80)	0.000
P13	0.567 (0.35–0.78)	0.000
P14	0.572 (0.37–0.78)	0.000
P15	0.549 (0.34–0.76)	0.000
P16	0.662 (0.48–0.85)	0.000
P17	0.398 (0.14–0.65)	0.000
P18	0.555 (0.34–0.77)	0.000
P19	0.612 (0.44–0.79)	0.000
P20	0.514 (0.32–0.71)	0.000
P21	0.644 (0.47–0.82)	0.000
P22	0.594 (0.43–0.76)	0.000
P23	0.674 (0.52–0.82)	0.000
P24	0.639 (0.40–0.87)	0.000
P25	0.612 (0.33–0.89)	0.000
P26	0.576 (0.34–0.82)	0.000
Overall	0.681 (0.64–0.72)	0.020

*CI 95%* Confidence interval 95%

### Questionnaire dimensions

An exploratory factor analysis was performed with Varimax rotation, obtaining a total of 7 dimensions that explain 65.9% of variability (Table 5).

**Table 5:** Component matrix with varimax rotation of the 26 items from the adapted SERVPERF questionnaire

Item	Factorial coefficients of the items after rotation						
	F1	F2	F3	F4	F5	F6	F7
p01	***0.696***	0.225	0.186	0.200	0.030	0.085	− 0.074
p02	***0.846***	0.102	0.131	0.122	0.080	− 0.016	0.015
p03	***0.593***	0.262	− 0.106	0.418	0.096	− 0.002	0.270
p04	***0.832***	0.138	0.093	0.086	0.018	− 0.017	0.114
p05	***0.758***	0.225	0.119	0.107	− 0.071	0.060	0.122
p06	***0.572***	0.155	0.448	0.292	− 0.003	− 0.016	− 0.001
p07	***0.584***	0.247	0.257	0.255	− 0.120	− 0.015	0.092
p08	0.245	***0.828***	0.145	0.003	0.050	0.053	0.022
p09	0.135	***0.838***	0.194	0.139	0.048	0.094	− 0.008
p10	0.262	***0.715***	0.153	0.254	− 0.005	0.000	0.040
p11	0.302	0.283	0.036	***0.617***	0.172	− 0.064	0.219
p12	0.329	*0.460*	***0.337***	0.086	0.020	− 0.002	− 0.062
p13	0.363	***0.453***	0.296	0.181	0.077	− 0.108	0.228
p14	0.086	0.330	***0.530***	0.187	0.112	− 0.014	0.187
p15	0.091	0.263	***0.792***	0.080	0.046	0.057	0.021
p16	0.254	0.109	***0.722***	0.083	0.006	0.100	0.191
p17	0.397	0.109	0.361	***0.579***	− 0.018	0.054	− 0.103
p18	0.263	0.202	0.255	***0.659***	0.077	0.146	− 0.046
p19	− 0.037	0.074	− 0.048	0.195	0.101	***0.785***	0.046
p20	0.050	0.033	0.027	0.083	0.431	***0.679***	0.122
p21	0.010	− 0.012	0.126	− 0.133	− 0.030	***0.731***	− 0.040
p22	− 0.010	0.042	0.076	0.152	***0.879***	0.010	− 0.047
p23	0.029	0.042	0.041	− 0.022	***0.806***	0.305	− 0.024
p24	***0.531***	0.142	− 0.092	− 0.354	0.368	− 0.158	0.161
p25	0.248	0.098	0.129	0.014	− 0.105	0.017	***0.780***
p26	− 0.018	− 0.041	0.104	0.017	0.045	0.059	***0.809***
Eigenvalues	8.088	2.379	1.743	1.482	1.332	1.104	1.008

Extraction method: Principal Component Analysis

Rotation method: Varimax normalization. The Kaiser criterion was used with an Eigenvalue > 1 to determine the resulting factors and explained variance (65.9%). In each dimension, those items that presented a saturation value > 0.40. The saturation of elements grouped in each dimension is shown bold and italics with the exception the item P12 in f2 is shown the non-bold italics explained in the Questionnaire dimensions section

Table 5 presents the factorial coefficients of the component matrix with Varimax rotation of the 26 items and Table 6 shows the dimensions in which the items are grouped, as well as the internal consistency values (Cronbach's alpha) for all items (0.862) and each dimension. All the items initially proposed for each dimension were maintained except for item 12, which was transferred from dimension 2 to 3 as this was a better conceptual fit. The decision was upheld because the alpha coefficients of dimensions 2 and 3 did not undergo significant changes (0.834 to 0.824 and 0.724 to 0.727, respectively).

**Table 6:** Dimensions resulting from the factor analysis, their internal consistency and list of associated items

Dimensions	Description	Cronbach’s alpha	Number of items	Associated items
1	Health professionals	0.861	8	1, 2, 3, 4, 5, 6, 7, 24
2	Administrative staff	0.824	4	8, 9, 10, 13
3	Organization management	0.727	4	12, 14, 15, 16
4	Information provided	0.737	3	11, 17, 18
5	Clinical aspects of the process	0.676	3	19, 20, 21
6	Impact of the process	0.749	2	22, 23
7	Satisfaction process	0.598	2	25, 26
Overall		0.862	26	1–26

In general, the internal consistency obtained for each dimension shows acceptable or good values, except for dimensions 5 and 7, in which it was somewhat low.

Thus, 5 dimensions similar to those of the SERVPERF scale were obtained, corresponding to healthcare personnel and administrative staff, management or tangible dimension, information and impact of the process, clinical aspects of the process (including pain, bleeding and anxiety), and satisfaction with the process, (would the patient recommend the process to a friend, would the patient come back if necessary). These last two dimensions, which were specific to the MA process, obtained the lowest internal consistency scores.

## Discussion

The results of the validation process of the adapted SERVPERF scale present a valid instrument for measuring satisfaction and quality of service in patients who request a MA.

The sociodemographic characteristics of the participants are similar to those of the population in Catalonia that requests a MA, according to the 2018 statistics reported by the Department of Health [5]. The most relevant difference is that 42% of patients were locals in the reported statistics, while in our study 66.3% were. This is most likely explained by having included improficiency in Spanish as an exclusion criterion.

Prior to this study, the effectiveness of the MA process had already been demonstrated and supported by protocols [5], but no data had been collected on quality as perceived by patients. In 2019, the first article on a validated, person-centered abortion care scale was published in Kenya [24]. The lack of data on the quality of abortion care may be due to the highly stigmatized status of the procedure.

McLemore assessed the experience of the outpatient abortion process in the United States: 70% of patients reported having had a better experience than expected; the rest mentioned the need to improve pain management and waiting time [21]. These findings support the decision to include 5 items related to the MA process in our proposal.

In 2020, Sudhinaraset et al. [25] published a validation of a person-centered abortion scale, in both surgical and medical private care, in a restrictive legal context of abortion. The dimensions of respectful care and communication predominated. They found that these types of scales can be adapted for different sexual and reproductive health services. Our scale also assesses the organization, clinical aspects and impact of the process.

Baynes studied how women experience post-MA visits in Tanzania [26]. Although the women were satisfied with the privacy and proximity of care, they identified significant areas for improvement: office cleanliness, post-contraception counseling, and pain management. In our study, the quality of these aspects was assessed as good. The scale presents good metric characteristics since it does not show saturated floor or ceiling effects and there was a high response rate for all items. The non-response rates for items 22–24 might be due to their placement on the back page of the questionnaire [23].

In general, the scores were high for all items, except for 19–23, which were related to the MA process. This is consistent with other studies in which items related to pain management, bleeding, and anxiety during the process scored lower [20, 21].

In the factor analysis, 7 dimensions were obtained that explain a total variance of 65.9%, similar to that obtained by Gómez-Besteiro (69.3%) [18].

The items added to address the process were grouped into two specific dimensions, which was deemed coherent.

In general, item agreement was moderate to excellent, except for items 6, 8 and 11, for which it was moderate, likely due to a certain degree of subjectivity. Predisposition, time dedicated and sufficient information may be perceived differently depending on patients’ need for support.

Item 17, which asked about the information provided to prevent unwanted pregnancies in the future, showed low reliability. This was also observed in the Baynes study as an aspect to be improved [26]. One solution would be to provide this information at the end of the process along with free contraception.

The dimensions obtained are similar to those proposed in other SERVPERF validation processes for healthcare. Gómez-Besteiro obtained the same 5 dimensions but distinguished between medical and nursing staff [18]. In our study, the healthcare professionals dimension included gynecologists and midwives, since both are involved in the process. Torres obtained 7 dimensions, including safety [27], which has already been analyzed in our area [5].

As for the limitations of this study, the important ethical-moral connotation of MAs must be considered. Although it is currently legal, it is still an ethical conflict. This factor may have influenced the number of study dropouts.

After performing the MA, some women did not attend follow-up visits. However, the dropout rate was low (13.2%) and no differences were observed that would suggest the existence of any type of risk.

## Conclusions

The results of this study provide a valid and reliable instrument for measuring the perception of quality in the service of users of a MA. With 26 items and a filling time of about 15 min, it makes it a useful and feasible tool for the continuous improvement of the service.

This scale is the best tool to assess and improve the quality of the MA service, with a view towards excellence in the sphere of public health.

## Data Availability

The data for the analysis, the Spanish questionnaires used and other supplementary material are available in Mendeley Data [23], 10.17632/45jz576dny.4.

## Abbreviations

MA: Medical Abortion
WG: Weeks Gestation
ASSIR: Sexual and Reproductive Health Care Centers
PC: Primary Care
WHO: World Health Organization
PLAENSA: Satisfaction survey plan
SERVPERF: SERVice-PERFormance
IDIAPJGol: Foundation University Institute for Primary Health Care Research Jordi Gol i Gurina
TeleForm: OpenText TeleForm, Data Capture Software
